# Treatment Resistant Severe Digital Ischemia Associated with Antiphospholipid Syndrome in a Male Patient with Systemic Sclerosis

**DOI:** 10.1155/2014/291382

**Published:** 2014-07-08

**Authors:** Orhan Küçükşahin, Aşkın Ateş, Alexis K. Okoh, Emre Kulahcioglu, Murat Turgay, Gülay Kınıklı

**Affiliations:** ^1^Department of Rheumatology, Ankara University Medical Faculty, Sihhiye, 06410 Ankara, Turkey; ^2^Department of Internal Medicine, Ankara University Medical Faculty, Sihhiye, 06410 Ankara, Turkey

## Abstract

We report the case of a male patient with limited cutaneous systemic sclerosis (SSc) that was complicated with severe digital ischemia, resistant to medical treatment. Due to the lack of treatment response, further laboratory and imaging studies were conducted. Findings were compatible with antiphospholipid syndrome and oral warfarin was added to the treatment regimen. After successful anticoagulation no further recurrences of digital ischemia were seen. An underlying etiology in SSc patients with treatment resistant digital ischemic necrosis should be suspected for accompanying antiphospholipid syndrome (APS).

## 1. Case Report

**Figure 1:**
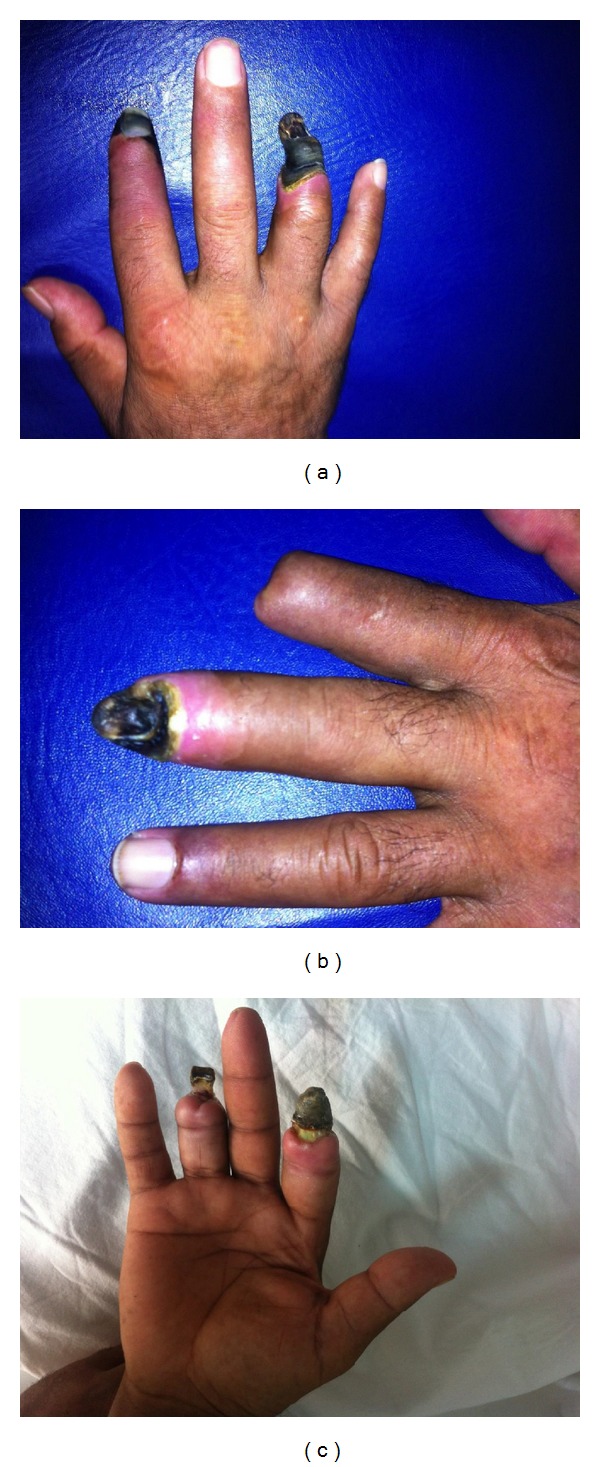
(a) Right hand showing digital gangrene on the index and fourth fingers, (b) left hand showing digital gangrene of third finger, and (c) six months after aggressive therapy.

A 44-year-old, previously well, nonsmoker male presented with severe digital ischemia of multiple fingers. Six months ago, due to digital gangrene, his distal left index finger was amputated. On examination, digital necroses of the distal right index, right fourth, and left third fingers were seen (Figures 1(a) and 1(b)). Investigations revealed the following: ANA (1/1000 centromeric pattern, +++) and anticentromere antibody were positive (1422 U/mL; normal: 0–10 U/mL) and erythrocyte sedimentation rate of 65 mm/hour, C-reactive protein of 92 mg/dL (normal: 0–3 mg/dL), thrombocytes of 100 000 (normal: 150–400 10^9^/L), and ANCA were negative. Nailfold capillaroscopy was consistent with an active scleroderma pattern (diffuse giant capillaries, microhemorrhages, and a reduction in number of capillaries). Chest radiograph, ECG, and HRCT findings of the thorax were normal. Esophagus motility test was normal as well. An upper extremity arterial Doppler ultrasound showed a normal flow pattern.

A diagnosis of limited cutaneous sclerosis was made. The patient was treated with acetylsalicylic acid 150 mg/day, pentoxifylline 1200 mg/day, nifedipine 60 mg/day, 400 mg/day hydroxychloroquine, and prednisolone 10 mg/day. Despite this therapy, he had severe attacks of Raynaud's phenomenon and newly developed digital ischemic ulcers. Two months later intravenous (IV) iloprost, bosentan, high-dose steroid, and IV cyclophosphamide were added.

**Figure 2:**
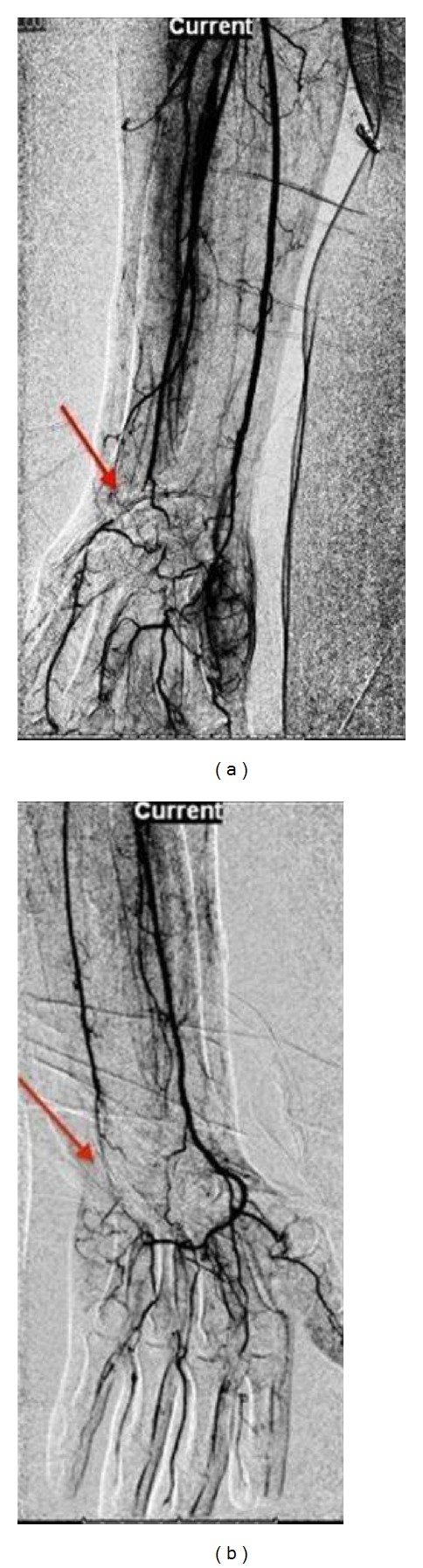
(a) Arrow showing total occlusion of the right radial and ulnar arteries at the wrist level. Filling of the radial artery seen after a short segment. (b) Arrow showing occlusion of the left ulnar artery at the wrist level with an open radial artery.

Due to the lack of treatment response further investigations were promptly done in order to define the underlying etiology of recurrent digital gangrene. Digital subtraction angiography (DSA) imaging of the upper extremity demonstrated occlusion of the left ulnar and right radial and ulnar arteries (Figure 2). Laboratory tests for APS showed LA: 1.4 (upper limit: 1.2) anti-Beta2 glycoprotein IgM: 9.3 MPL U/mL (0–5), anticardiolipin IgM: 7.5 MPL U/mL (0–5), and thrombocytopenia. Triggered by the suspicion of an underlying possible infection, the patient was tested for syphilis, leptospirosis, Lyme disease, hepatitis B and C viruses, and human immunodeficiency virus (HIV); however results from infectious diseases panel were unremarkable. A diagnosis of an overlap syndrome, SSc, associated with antiphospholipid syndrome was made and oral warfarin was added to his treatment regimen.

Capillaroscopy performed on his follow-up visit six months later revealed findings (right 2, 3, and 5 fingers; 2-3 non angiogenic capillaries; absent giant capillaries and microhemorrhages, left 5 and 6 fingers; and 5-6 microhemorrhages) consistent with late stage scleroderma.

On current treatment including oral daily use of bosentan, sildenafil, hydroxychloroquine, acetyl salicylic acid, warfarin, and monthly IV cyclophosphamide over six months of follow-up, the patient remained well, with no further recurrences of digital ischemic lesions (Figure 1(c)).

## 2. Discussion

The coexistence of SSc/APS is a very rare overlap syndrome, found in less than 1% of scleroderma patients, yet the positivity for antiphospholipid antibodies in SSc is higher [1, 2]. When it is seen, it is associated with more severe manifestations including digital infarct, gangrene, and pulmonary hypertension [3]. Minatani et al. previously reported the first presentation of this overlap syndrome in a male patient [4]. One case series with five female patients [5] and other three cases of female patients [6–8] were also reported in the literature.

Treatment with bosentan or its combination with sildenafil has been reported to decrease the number of new ulcers in systemic sclerosis [9, 10]. Ambach et al. reported resolution of digital ulcers in a 73-year-old female patient who had uncontrolled disease over a 10-year period after a combination therapy of bosentan and sildenafil [10].

Surprisingly, despite treatment with a range of immunosuppressive therapies and vasodilators, this patient's digital ischemia continued to progress. The patient was treated with methylprednisolone and cyclophosphamide considering the fact that small vessel vasculitis potentially contributed to the microangiopathy. Blood pressure and renal function were monitored carefully for the risk of scleroderma renal crisis. Despite the combination of various therapeutic agents, over 2 months, we failed to halt the progression of digital ischemia.

After reconsideration of the causes of underlying recurrent, treatment resistant digital gangrene, an overlap syndrome, SSc, associated with antiphospholipid syndrome was diagnosed with further laboratory and imaging studies; oral warfarin was added to the treatment regimen. Inevitably, the infarcted tissue was amputated, but over a year of follow-up, the patient remained well, with no further recurrences of digital ischemic lesions.
